# The effect of exercise therapy as a tool for preventing and treating musculoskeletal disorders among school-aged children: a randomised controlled trial

**DOI:** 10.1186/s12891-024-07510-1

**Published:** 2024-05-14

**Authors:** Farhad Shourie, Behnam Ghasemi, Ali Shafizadeh, Sajad Bagherian, Evert Verhagen

**Affiliations:** 1 Department of Sport Sciences, Shahrekord University, Shahrekord, Iran; 2 Department of Public and Occupational Health, Location Vrije Universiteit, Amsterdam University Medical Centers, Amsterdam, Netherlands

**Keywords:** Musculoskeletal pain, Corrective exercise, Physical activity, Quality of life

## Abstract

**Background:**

Children spend a lot of time in school, and there are many ergonomic hazards and postural malalignments that put children at greater risk of developing musculoskeletal disorders (MSDs). This study aimed to investigate the effect of exercise therapy on preventing and treating musculoskeletal disorders among school-aged children.

**Method:**

This randomised controlled trial included 212 (121 boys and 91 girls) school-aged children aged 13–15 years assigned to treatment (*n* = 106) and prevention (*n* = 106) groups, where the treatment group contained individuals with MSDs and prevention group contained individuals without MSDs. In each group, half of the individuals received exercise therapy (50 min per session, four times per week, for an 8-week), and others continued their daily lives. MSDs and physical activity were assessed by the Teen Nordic Musculoskeletal Screening Questionnaire and the International Physical Activity Questionnaire-Short Form, respectively, at baseline and after the experimental protocol.

**Results:**

There was a statistically significant reduction in the frequency of MSDs in the treatment group and occurring MSDs in the prevention group (*P* ≤ .05). Also, there was significant improvement in all variables of walking, moderate physical activity, vigorous physical activity, and total in intervention groups spatially in students who received exercise therapy (*P* ≤ .05).

**Conclusions:**

This study demonstrated the effectiveness of exercise therapy in reducing and preventing MSDs and improving physical activity levels among school-aged children aged 13–15 years.

**Trial registrations:**

Ethical Committee of Shahrekord University (IR.SKU.REC.1401.022) (registration date: 31/05/2022). Clinical Trail Registration (IRCT20220705055375N1), (registration date: 29/07/2022).

## Background

Globally, the most frequent cause of chronic pain and physical disability is musculoskeletal disorders (MSDs) [1]. They are abnormalities in the body’s structures—muscles, tendons, ligaments, joints, nerves, and supporting blood vessels—that cause discomfort, aches, or numbness in the limbs—upper and lower—or in the region surrounding the spine [2]. These conditions vary from person to person and worsen with ageing and recurrent injuries. They can cause mild physical impairments, pain, or discomfort and potentially impair a person’s ability to carry out daily tasks [3]. They are brought on by prolonged postures and repeated movements that affect the properties of the tissue, which ultimately alters the pattern of movement and, in less-than-perfect circumstances, can result in MSDs [4].

Children must sit for extended amounts of time in the existing educational system, and the more technology used in the classroom, the more hours they must spend sitting [5]. Prolonged sitting has been connected to the emergence of back pain [6]. In addition, throughout their virtual learning experiences, students who engage in online or remote learning may adopt various habitual postures (such as slouching when sitting, lying down or in a prone position, or sitting on the edge of a chair) [7]. These positions reflect improper alignment of the body that results in micro spasms, a reduction in the soft tissue’s natural elasticity, and modifications to the length and strength of muscles that affect the relative involvement of the antagonists and synergists, all contributing to MSDs [4]. Students often endure physical strain during their academic pursuits due to their bad posture when sitting, standing, or both. This can result in joint instability and muscular tension, which, over time, can cause chronic and recurrent episodes of discomfort [8].

Children and adolescents who engage in physical exercise tend to have better health results, whereas those who spend too much time in sedentary activities have worse health outcomes [9]. Consequently, the World Health Assembly set two targets: a global reduction in physical inactivity of 10% by 2025 and a global reduction of 15% by 2030 [10]. The World Health Organization (WHO) advises that people aged 5 to 17 participate in at least 60 min of moderate-to-vigorous physical activity each day of the week because regular physical activity benefits a person’s health [11]. Furthermore, engaging in aerobics, high-intensity training, and bone and muscle strengthening exercises three days a week Field [10] is recommended. Regular physical activity can help fulfil job requirements and improve quality of life, regardless of age. Additionally, leisure-time physical activity can lower the risk of MSDs in people of all ages [12]. While regular activity offers numerous benefits, incorporating targeted training methods can further enhance results. For example, Pilates, a widely implemented exercise program in schools, has proven highly effective in preventing back pain, as evidenced by the Patti et al. study [13].

According to earlier research, young individuals’ physical activity declines as they approach early adulthood, with the most considerable reduction occurring when they are accepted into a university [14]. It has been demonstrated that therapeutic exercise programs and physical activity are beneficial in lowering musculoskeletal pain [15]. Exercise has been shown to reduce impairment, alleviate symptoms, and enhance the quality of life in various chronic musculoskeletal pain problems affecting professionals in multiple fields. There is little data to determine how exercise therapy affects the prevention and treatment of MSDs in school-age children, even though it is consistently beneficial. Therefore, this study was designed to investigate the effect of exercise therapy on preventing and treating MSDs among school-aged children aged 13–15 years. It was hypothesised that exercise therapy would treat and prevent MSDs when compared with baseline measures.

## Materials and methods

### Design of the study

**Fig. 1:**
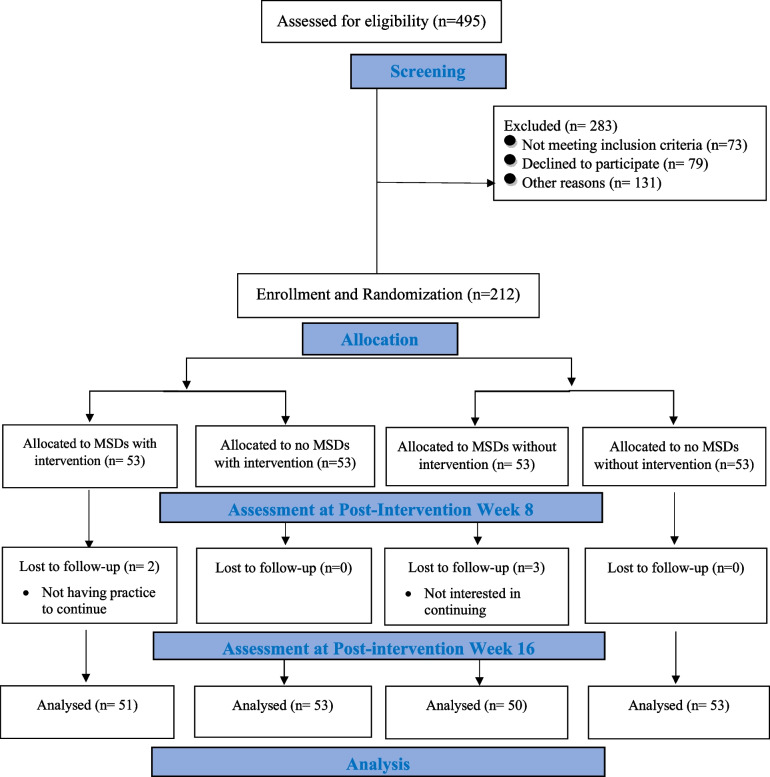
The Consolidated Standards of Reporting Trials (CONSORT) flow diagram of trial enrolment and follow-up

The study design was a parallel-group, randomised, controlled, assessor-blinded clinical trial. The intervention period was eight weeks, with pre-intervention baseline measurements and post-intervention follow-up measurements. All participants volunteered, and written informed consent was obtained from the participant’s parents or guardians. Participants could leave the study if they were reluctant to continue the trial. The trial was approved by the Ethics Committee of Shahrekord University (IR.SKU.REC.1401.022) (registration date: 31/05/2022). The trial was registered on the Iranian Clinical Trial Registry with identification number IRCT20220705055375N1 (registration date: 29/07/2022). This study conforms to all CONSORT guidelines and reports the required information accordingly [16]. The CONSORT flowchart is presented in Fig. 1.

### Participants and randomisation

We recruited students by contacting schools and discussing the project and its requirements with them. Students’ MSDs were then assessed as a screening strategy to identify those who have MSDs and those who do not. Participation in the study was voluntary, and all students attending local high schools could access this study. Participant inclusion criteria were as follows: aged 13–15 years, not having a health condition that could limit the ability to move, being able to perform a moderate-intensity exercise session and abstaining from all physical activity not involving the study protocol during the entire intervention period. The exclusion criteria were having an orthopaedic condition limiting the ability to perform an exercise program and not being able to abstain from all physical activities outside the study protocol during all the intervention days.

Also, MSDs of student who wanted to participate in study was assessed as a screening strategy to know who are have MSDs and who do not.

In simple randomisation with a 1:1 allocation ratio, 212 school-aged children (121 boys and 91 girls) aged 13–15 were randomly divided into two groups, 106 and selected using a computer-generated list of random numbers. Each intervention (*n* = 106) and control (*n* = 106) group contain individuals with MSDs (*n* = 53) and without MSDs (*n* = 53). In the intervention and control groups, there were two subgroups, including individuals with MSDs, called the “treatment group”, and individuals without MSDs, called the “prevention group”. The process of recruitment and allocation is described in Fig. 1.

### Sample size

The necessary sample size was estimated using G*Power 3.1.7 for Windows (G*Power©, University of Dusseldorf, Germany). To obtain 80% statistical power, an α error = 0.05, repeated-measure analysis of variance (ANOVA), and a medium effect size of 0.25 to consider four groups and two measurements for the primary outcome, generating a sample size of about 40 participants per group (total sample size of 160 subjects), considering a 10% dropout rate, the sample was increased to 200 (50 in each group).

### Procedure

All participants were methodically informed of the study details. MSDs and physical activity were assessed at baseline and postintervention for each group. All tests were performed by the same examiner, blinded to the group assignments and previous measurements. An expert corrective exercise specialist supervised all assessments.

## Outcome measures

### Musculoskeletal disorders

The symptom prevalence data was collected using the Teen Nordic Musculoskeletal Screening Questionnaire (TNMQ-S), a translated and adapted form of the Extended Nordic Musculoskeletal Questionnaire (NMQ-E) [17]. Legault et al. assessed the validity and reliability of the TNMQ-S in their study, demonstrating its effectiveness for measuring musculoskeletal symptoms in adolescents [18]. The TNMQ-S comprises three dichotomous questions over nine anatomical regions: the 6-month prevalence of musculoskeletal symptoms, the impact of these symptoms on school and work attendance, and their impact on sports and leisure activities. The anatomical regions in the TNMQ-S are the neck, shoulders, upper back, elbows, wrists and hands, lower back, hips and thighs, knees, and ankles and feet.

### Physical activity

The International Physical Activity Questionnaire-Short Form (IPAQ-SF) was used to assess the level of physical activity and sedentary behaviour of school-aged children before participating in the study [19]. It comprises four domains intended to measure the average duration devoted to walking and moderate- and vigorous-intensity. The weekly MET minutes were calculated by multiplying the MET factor assigned to each activity (walking = 3.3 MET, moderate-intensity activity = 4.0 MET, vigorous-intensity activity = 8.0 MET) by the duration (in minutes) and the number of days the respective activity was performed. Total physical activity was the sum of weekly MET minutes spent on walking and moderate- and vigorous-intensity activities [19]. IPAQ-SF showed good test retest reliability (ICC = 0.9) [20].

### Intervention

Following random assignment, students were divided into two groups: a treatment group (*n* = 53) consisting of students with MSDs, and a prevention group (*n* = 53) comprised of students without MSDs. Both groups participated in a moderate-intensity exercise intervention for 32 sessions (four days per week for eight weeks), with each session lasting approximately 50 min.

The intervention included a structured workout routine. Each session began with a 5–10 min warm-up consisting of light cardio and dynamic stretches to prepare the body for exercise. The main training session, lasting 30–40 min, focused on mobility, stretching, strengthening, and endurance. Perceived exertion during training was monitored using the Borg Rating of Perceived Exertion (RPE) Scale, with a target range of 12–14 (somewhat hard to hard) to ensure moderate-intensity exercise. The workout concluded with a 5–10 min cool-down focused on static stretches.

The rest time between each set was 1:3, and between repetitions was 1:1. The participants in each experimental group performed the intervention in a school’s sports facilities. Two corrective exercise specialists supervised them. The progression of the exercises was prescheduled but flexible according to everyone’s progression and limitations. Details of the sets and the repetitions are presented in Table 1 and 2.

**Table 1:** The exercise therapy program

Week 1–2	Instruction	Volume
Neck mobility	Bending and pulling left—right and front—back of the neck while standing	10r × 3 sets
Neck stretch	Stretch the neck to the sides by hand	Hold each side for 5 s × 3 set
Strengthen the neck	Resistance to neck movement by hand	Hold each side for 5 s × 3 set
Shoulder mobility	Ups and downs, shoulder rotation	10r × 3 sets
Shoulder stretch	Stretch the Shoulder to thesides by hand	20 s × 3 sets
Modified push-up	Lying on your abdomen and bending your knees	5r × 3 sets
Sit-up	Lie on your back, knees bent	15r × 3 sets
Cat & camel	Start moving on the arms and legs	20 s × 3 sets
Quadruped exercise	Raising the arm and contralateral leg	Hold each side for 10 s × 3 set
Pelvic mobility	Ups and downs in the supin	10r × 3 sets
Hamstring stretch	Lie on your back and raise one leg	20 s × 3 sets
Front Plank	Lie prone on mat or floor	20 s × 3 sets
Week 3–4	**Instruction**	**Volume**
Neck mobility	Bending and pulling left—right and front—back of the neck while standing	15r × 3 sets
Neck stretch	Stretch the neck to the sides by hand	Hold each side for 10 s × 3 set
Strengthen the neck	Resistance to neck movement by hand	Hold each side for 10 s × 3 set
Shoulder mobility	Ups and downs, shoulder rotation	15r × 3 sets
Shoulder stretch	Stretch the Shoulder to thesides by hand	25 s × 3 sets
Modified push-up	Lying on your abdomen and bending your knees	10r × 3 sets
Sit-up	Lie on your back, knees bent	20r × 3 sets
Cat & camel	Start moving on the arms and legs	25 s × 3 sets
Quadruped exercise	Raising the arm and contralateral leg	Hold each side for 15 s × 3 set
Pelvic mobility	Ups and downs in the supin	15r × 3 sets
Hamstring stretch	Lie on your back and raise one leg	25 s × 3 sets
Front Plank	Lie prone on mat or floor	25 s × 3 sets
Week 5–6	**Instruction**	**Volume**
Neck mobility	Bending and pulling left—right and front—back of the neck while standing	20r × 3 sets
Neck stretch	Stretch the neck to the sides by hand	Hold each side for 15 s × 3 set
Strengthen the neck	Resistance to neck movement by hand	Hold each side for 15 s × 3 set
Shoulder mobility	Ups and downs, shoulder rotation	20r × 3 sets
Shoulder stretch	Stretch the Shoulder to thesides by hand	30 s × 3 sets
Modified push-up	Lying on your abdomen and bending your knees	15r × 3 sets
Sit-up	Lie on your back, knees bent	25r × 3 sets
Cat & camel	Start moving on the arms and legs	30 s × 3 sets
Quadruped exercise	Raising the arm and contralateral leg	Hold each side for 20 s × 3 set
Pelvic mobility	Ups and downs in the supin	20r × 3 sets
Hamstring stretch	Lie on your back and raise one leg	30 s × 3 sets
Front Plank	Lie prone on mat or floor	30 s × 3 sets
Week 7–8	**Instruction**	**Volume**
Neck mobility	Bending and pulling left—right and front—back of the neck while standing	25r × 3 sets
Neck stretch	Stretch the neck to the sides by hand	Hold each side for 20 s × 3 set
Strengthen the neck	Resistance to neck movement by hand	Hold each side for 20 s × 3 set
Shoulder mobility	Ups and downs, shoulder rotation	25r × 3 sets
Shoulder stretch	Stretch the Shoulder to thesides by hand	40 s × 3 sets
Modified push-up	Lying on your abdomen and bending your knees	20r × 3 sets
Sit-up	Lie on your back, knees bent	30r × 3 sets
Cat & camel	Start moving on the arms and legs	40 s × 3 sets
Quadruped exercise	Raising the arm and contralateral leg	Hold each side for 20 s × 3 set
Pelvic mobility	Ups and downs in the supin	25r × 3 sets
Hamstring stretch	Lie on your back and raise one leg	40 s × 3 sets
Front Plank	Lie prone on mat or floor	40 s × 3 sets

*r* Repetition, *s* Second

**Table 2:**
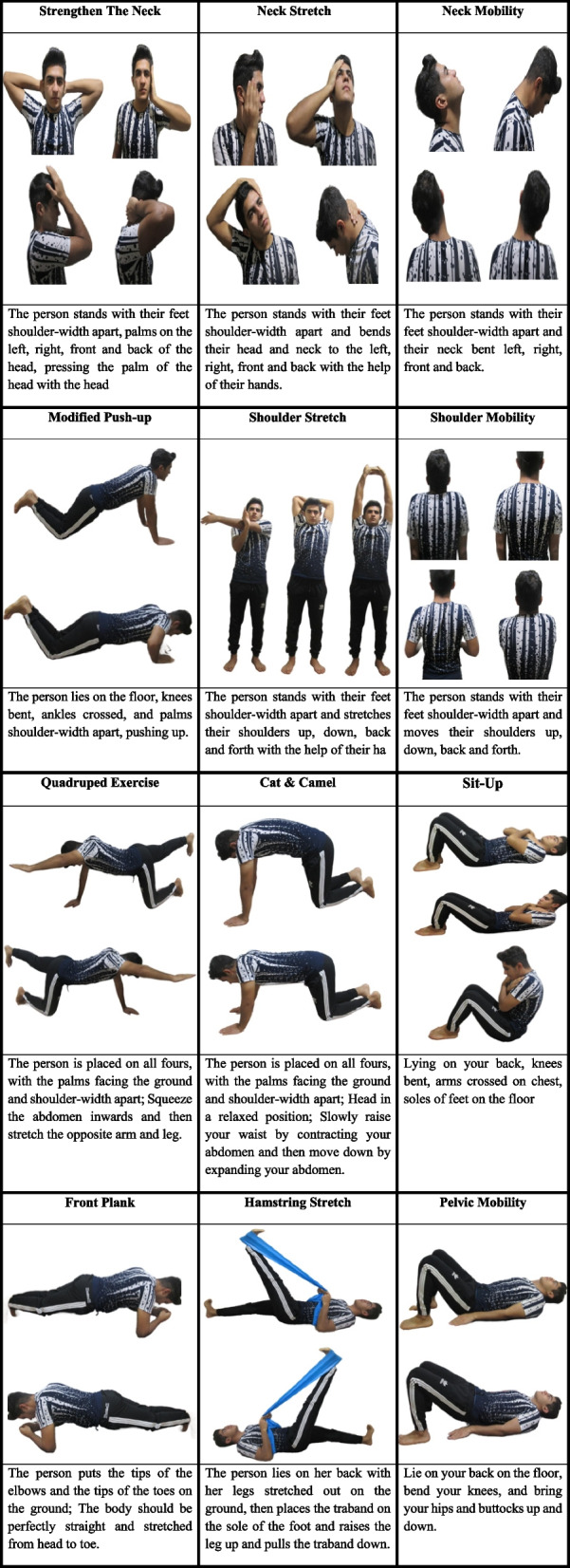
The exercise therapy program

### Statistical analyses

Statistical analysis was done using the SPSS 26.0 version (IBM Corporation, Armonk, NY, USA). The significance levels were set at alpha < 0.05 for all analyses. Results from a Shapiro–Wilk test revealed that the data were not normally distributed; thus, to analyse the results concerning pain in the different body areas before and after the intervention, a comparison of proportions was conducted using the Chi‑square (χ^2^) test. The chi-square test examines whether there is an association between two categorical variables in this study: the time (pre- and post-test) and whether the MSDs were treated. Cramer’s V (V_Cramer_) was used to indicate the strength of association in chi-square tests for nominal variables. The value of Cramer’s V can range from 0 to 1, with 0 indicating no association and 1 indicating a perfect association. Effect sizes of 0.10, 0.30, and 0.50 were considered small, medium, and large effects, respectively [21].

Regarding physical activity energy expenditure in MET-minutes/week, the mean difference was tested with the Wilcoxon signed rank test. The effect sizes were calculated using Cohen’s d and interpreted as weak (< 0.2), small (0.21–0.5), medium (0.51–0.8), or large (> 0.8).

## Results

The baseline characteristics of the study participants are summarised in Table 3. Our results have shown that before randomisation and dividing schoolchildren into groups, the prevalence of MSDs was high in students who had MSDs: neck pain (35%), shoulder pain (35%), upper back pain (35%), elbow pain (26%), wrists/hands pain (28%), lower back pain (22%), hips/tights pain (12%), knee pain (32%), and ankle pain (34%). Also, the prevalence of MSDs in schoolchildren after randomisation is shown in Table 4.

**Table 3:** Baseline characteristics of the study participants. Values are given as mean ± SD

Index	Treatment group		Prevention group	
	**Students with MSDs received Exercise (*n* = 51)**	**Control with MSDs (*n* = 53)**	**Students without MSDs received Exercise (*n* = 50)**	**Control without MSDs (*n* = 53)**
Age (year)	14.2 ± 0.71	13.9 ± 0.67	13.9 ± 0.65	13.9 ± 0.66
Height (cm)	161.7 ± 13.7	158.9 ± 12.7	163.7 ± 10.3	160.2 ± 15.1
Weight (kg)	52.2 ± 14.6	55.8 ± 14.4	57.38 ± 13.7	53.7 ± 12.1
BMI	19.8 ± 4.31	22.1 ± 5.1	21.2 ± 4.03	21.1 ± 5.01

**Table 4:** Prevalence of MSDs in groups of treatment and prevention in two times of measurements

		**Treatment group**		**Prevention** **group**	
		**Students with MSDs received Exercise **(*n*=51)	**Control with MSDs **(*n*=53)	**Students without MSDs received Exercise** (*n*=50)	**Control without MSDs **(*n*=53)
**Neck pain**	Pre-test	14 (27.5ا%)*	21 (42%)	0 (00%)	0 (00%)*
	Post-test	1 (2.0%)	23 (46%)	0 (00%)	7 (13.2%)
	% Change	-92%	9%	0	-
	Chi‑square	χ^2^=13.2, *P*=0.000, V_Cramer_=0.36	χ^2^=0.16, *P*=0.68, V_Cramer_=0.04	-	χ^2^=7.5, *P*=0.006, V_Cramer_=0.27
**Shoulder pain**	Pre-test	14 (27.5%)*	21 (42%)	0 (00%)	0 (00%)*
	Post-test	0 (0.0%)	26 (52%)	0 (00%)	7 (13.2%)
	% Change	-100%	23%	0	-
	Chi‑square	χ^2^=16.2, *P*=0.000, V_Cramer_=0.40	χ^2^=1.1, *P*=0.32, V_Cramer_=0.10	-	χ^2^=7.5, *P*=0.006, V_Cramer_=0.27
**Upper back pain**	Pre-test	14 (27.5%)*	21 (42%)	0 (00%)	0 (00%)*
	Post-test	1 (2.0%)	24 (48%)	0 (00%)	6 (11.3%)
	% Change	-92%	14%	0	-
	Chi‑square	χ^2^=13.2, *P*=0.000, V_Cramer_=0.36	χ^2^=0.62, *P*=0.43, V_Cramer_=0.08	-	χ^2^=6.4, *P*=0.012, V_Cramer_=0.25
**Elbows pain**	Pre-test	12 (23.5%)*	14 (28%)	0 (00%)	0 (00%)
	Post-test	1 (2.0%)	12 (24%)	0 (00%)	4 (7.5%)
	% Change	-91%	-14%	0	-
	Chi‑square	χ^2^=10.67, *P*=0.001, V_Cramer_=0.32	χ^2^=0.21, *P*=0.65, V_Cramer_=0.05	-	χ^2^=4.2, *P*=0.041, V_Cramer_=0.19
**Wrists hands pain**	Pre-test	10(19.6%)*	18 (36%)	0 (00%)	0 (00%)
	Post-test	0 (0.0%)	14 (28%)	1 (1.9%)	1 (1.9%)
	% Change	-100%	-22%	-	-
	Chi‑square	χ^2^=11.1, *P*=0.001, V_Cramer_=0.33	χ^2^=0.52, *P*=0.47, V_Cramer_=0.07	χ^2^=0.94, P=0.333, V_Cramer_=0.09	χ^2^=0.94, *P*=0.333, V_Cramer_=0.09
**Lower back pain**	Pre-test	6 (11.8%)*	16 (32%)	0 (00%)	0 (00%)
	Post-test	0 (0.0%)	19 (38%)	0 (00%)	4 (7.5%)
	% Change	-100%	18%	0	0
	Chi‑square	χ^2^=6.4, *P*=0.012, V_Cramer_=0.25	χ^2^=0.39, *P*=0.53, V_Cramer_=0.06	-	χ^2^=4.2, *P*=0.041, V_Cramer_=0.19
**Hips tights pain**	Pre-test	4 (7.8%)	8 (16%)	0 (00%)	0 (00%)
	Post-test	0 (0.0%)	8 (16%)	0 (00%)	3 (5.7%)
	% Change	-100%	0	0	-
	Chi‑square	χ^2^=4.2, *P*=0.041, V_Cramer_=0.20	-	-	χ^2^=3.1, *P*=0.08, V_Cramer_=0.17
**Knees pain**	Pre-test	16 (31.4%)*	16 (32%)	0 (00%)	0 (00%)*
	Post-test	2 (3.9%)	14 (28%)	0 (00%)	7 (13.2%)
	% Change	-87%	-12%	0	-
	Chi‑square	χ^2^=13.2, *P*=0.001, V_Cramer_=0.36	χ^2^=0.13, *P*=0.711, V_Cramer_=0.04	-	χ^2^=7.5, *P*=0.006, V_Cramer_=0.27
**Ankle pain**	Pre-test	19 (37.3%)*	15 (30%)	0 (00%)	0 (00%)
	Post-test	2 (3.9%)	16 (32%)	0 (00%)	3 (5.7%)
	% Change	-89%	6%	0	-
	Chi‑square	χ^2^=17.3, *P*=0.001, V_Cramer_=0.41	χ^2^=0.05, *P*=0.829, V_Cramer_=0.02	-	χ^2^=3.1, *P*=0.079, V_Cramer_=0.17

*Statistically significant change in score from baseline to posttest (*P*<0.05).

In the treatment group, there was a statistically significant reduction in the frequency of MSDs between pre-intervention baseline measurements and post-intervention follow-up measurements in students with MSDs who received exercise therapy in all body regions, as shown in Table 4 (*P* ≤ 0.05). In the prevention group, where students who did not have MSDs participated and received exercise therapy, there were no changes in MSDs (*P* > 0.05), which means exercise therapy works well for preventing MSDs (Table 4).

Table 5 shows the pre-post group comparison for total weekly energy expenditure in MET minutes/week. There was significant improvement in all variables of walking, moderate physical activity, vigorous physical activity, and total in intervention groups spatially in students who received exercise therapy (*P* ≤ 0.05) (Table 5).

**Table 5:** Total weekly energy expenditure in MET-minutes/week.

		**Treatment group**		**Prevention** **group**	
		**Students with MSDs received Exercise (*n*=51)**	**Control with MSDs (*n*=53)**	**Students without MSDs received Exercise (*n*=50)**	**Control without MSDs (*n*=53)**
**Walking**	Pre-test	328.64±202.91	316.73±230.63	319.1±164.36	281.12±176.91
	Post-test	455.52±193.27	301.62±171.11	423.7±150.97	251.85±168.83
	% Change	38%	-4%	32%	-10%
	*P* value & Cohen d	z= -3.7, *P*<0.001 , d=0.64	z= -.54, *P*=0.58 , d=.07	z= -4.6, *P*<0.001 , d=0.66	z= -3.2, *P*<0.001 , d=0.16
**Moderate physical activity**	Pre-test	268.62±276.67	240.8±259.44	237.88±222.08	174.33±164.66
	Post-test	397.65±197.0	191.6±179.7	390.19±220.09	198.87±152.23
	% Change	48%	-20%	64%	14%
	P value & Cohen d	z= -3.1, *P*=0.002 , d=0.53	z= -.41, *P*=.67 , d=0.22	z= -4.1, *P*<0.001 , d=0.68	z= -.94, *P*=0.34 , d=0.15
**Vigorous physical activity**	Pre-test	181.96±363.38	169.6±300.04	241.50±325.8	160.75±218.03
	Post-test	521.25±373.82	39.2±89.43	544.91±440.43	64.91±178.75
	% Change	186%	-76%	125%	-59%
	*P* value & Cohen d	z= -5.02, *P*<0.001 , d=0.92	z= -2.99, *P*=0.003 , d=0.58	z= -3.7, *P*<0.001 , d=0.78	z= -3.2 *P*<0.001, d=0.48
**Total**	Pre-test	779.27±601.31	727.24±517.7	798.6±449.94	616.91±328.1
	Post-test	1374.51±504.81	532.48±268.0	1358.9±442.84	515.68±287.78
	% Change	76%	26%	282%	16%
	*P* value & Cohen d	z= -5.37, P<0.001 , d=1.07	z= -2.89, *P*=0.004 , d=0.47	z= -4.6, *P*<0.001 , d=0.86	z= -2.4, *P*=0.015 , d=0.32

Mean difference was tested with the Wilcoxon Signed-Rank Test. Significance was considered at *p* < 0.05

## Discussion

The study’s first aim was to investigate the effect of exercise therapy on preventing and treating MSDs among school-aged children aged 13–15 years. According to the results of our study, the treatment group showed a reduction in the frequency of MSDs, and the prevention group showed no MSDs in pre- and post-treatment comparisons. These results magnify the significant effect of exercise therapy on the reduction and prevention of MSDs in school-aged children. The study results reflect that the frequency of MSDs decreased in the treatment group and that MSDs were prevented in the prevention group. The study’s results complement previous studies that show that exercise therapy reduced MSDs in different occupations [22, 23].

The second aim was to investigate the effect of exercise therapy on physical activity levels among school-aged children aged 13–15 years. Our results showed that the level of physical activity in intervention groups in comparison to baseline improved spatially in a group of students with MSDs who received exercise therapy. These results highlight the significant effect of exercise therapy on increasing physical activity in school-aged children. The study’s results agree with previous studies’ results [24, 25].

Prolonged sitting is a growing concern for children’s health, contributing to MSDs. Our findings align with existing research [26] highlighting a high prevalence of MSDs in schoolchildren, particularly affecting the neck. This coincides with children spending a significant portion of their day in seated positions at school (average 5 h) and using computers at home (1.5 h) [26]. While sitting time is a factor, research suggests that posture plays a more critical role. Prolonged sitting with improper neck, back, and torso alignment (flexed or twisted) is linked to an increased risk of MSDs [9]. Ergonomic interventions in schools can mitigate these issues. These interventions go beyond simply encouraging breaks and include established techniques like active breaks and learning through movement, alongside natural movement projects. Active breaks involve short bursts of physical activity, such as jumping jacks or stretches, dispersed throughout the school day, and have been shown to improve student focus and concentration [27]. Learning through movement builds on this concept, integrating highly relevant physical activities directly linked to learning material (e.g., reenacting historical events, kinesthetic math problems) [28]. Natural movement projects can further foster student engagement by encouraging them to integrate physical activity into their learning projects in creative ways. This might involve creating a dance representing a scientific concept or constructing a model requiring physical construction [29]. Implementing a combination of these techniques can foster a more engaging and dynamic learning environment, potentially leading to a range of benefits for children’s health and learning.

However, it is well-recognised that regular exercise promotes better physical health. For example, Herbert et al. [30] demonstrated that regular physical activity might improve university students’ physical health. A Munchaona study’s findings showed that, compared to the control group, the experimental group’s musculoskeletal pain decreased by the intervention [31]. Our study evaluated the prevalence of MSDs and physical activity levels and the effect of exercise therapy interventions on these variables. According to a different study, children and adolescents gain weight over the summer, as was previously noted, and the number of months that schools are closed may correlate with an increase in children’s obesity rates [32]. A healthy body weight, a healthy cardiovascular system (heart and lungs), neuromuscular awareness (control and coordination of movement), and musculoskeletal tissues (muscles, bones, and joints) can all be developed with appropriate amounts of physical activity [33]. The majority of children and adolescents worldwide do not fulfil the recommended levels of physical activity despite the advantages of physical activity being widely recognised [34]. Research by Bonavolontà et al. [35] suggests that people are more likely to continue activities they find enjoyable. This is because positive experiences can lead to increased motivation and a desire to repeat the behavior, potentially forming a habit [35].

Most sedentary people’s days are spent sitting or lying down, reading, talking, watching TV, or using a computer or phone to take virtual classes. The increasing usage of electronics and related keyboard activity makes it difficult for people to maintain proper posture and engage in physical activity [36]. Research indicates that treating musculoskeletal issues and postural difficulties brought on by excessive technology use is becoming increasingly important [36]. Sufficient data suggests that children’s postural patterns must be monitored and improved [9]. Muscle misalignment, or bad posture, can change the length of the muscle, which reduces tension development and makes the muscle unable to produce enough force for an efficient and effective movement [37]. A comprehensive evaluation found a connection between sitting position and upper quadrant musculoskeletal discomfort in children and adolescents [38]. The study’s findings advance our knowledge of how exercise therapy programs affect physical activity levels and MSDs. The study’s practical implications will focus on how exercise therapy protocols can treat and prevent MSDs and improve the physical activity levels of school-age students at risk of physical inactivity and MSDs due to today’s lifestyle and daily activity postural patterns. The main strengths of this study are the relatively large number of participants in groups who met the inclusion criteria and the age of the participants, who were school-age students. Furthermore, as our study is one of the few on school children available in the global literature, it may impact future research and offer valuable information.

### Limits of the study

While questionnaires provided valuable insights, the reliance on self-reported data presents a potential limitation. Future studies could utilize objective measures like accelerometers to obtain a more precise and unbiased assessment of participants’ physical activity levels. This study did not collect information about participants’ background characteristics, including physical background (e.g., health status, fitness level, and sport background) and cultural and social background (of both children and parents). This omission limits the generalizability of the results, as factors like health, fitness, prior sport experience, cultural values, and socioeconomic status can influence the outcomes of the study. Considering these findings and the limitations of the current study, more research is necessary to validate our findings in a more homogeneous group and on a bigger scale.

## Conclusions

The prevalence of musculoskeletal pain, particularly chronic pain, in schoolchildren aged 13–15 years was high. The results of this study demonstrated the effectiveness of exercise therapy in reducing and preventing MSDs and improving physical activity levels among school-aged children aged 13–15 years.

## Data Availability

The dataset analysed for this study is available from the corresponding author on reasonable request.
